# Absence of adaptive evolution is the main barrier against influenza emergence in horses in Asia despite frequent virus interspecies transmission from wild birds

**DOI:** 10.1371/journal.ppat.1007531

**Published:** 2019-02-07

**Authors:** Henan Zhu, Batchuluun Damdinjav, Gaelle Gonzalez, Livia Victoria Patrono, Humberto Ramirez-Mendoza, Julien A. R. Amat, Joanna Crispell, Yasmin Amy Parr, Toni-ann Hammond, Enkhtuvshin Shiilegdamba, Y. H. Connie Leung, Malik Peiris, John F. Marshall, Joseph Hughes, Martin Gilbert, Pablo R. Murcia

**Affiliations:** 1 MRC-University of Glasgow Centre for Virus Research, Glasgow, United Kingdom; 2 State Central Veterinary Laboratory, Transboundary Animal Disease Laboratory, Avian Influenza Section, Ulaanbaatar, Mongolia; 3 Project Group Epidemiology of Highly Pathogenic Microorganisms, Robert Koch Institute, Berlin, Germany; 4 Departamento de Microbiología e Inmunología, Facultad de Medicina Veterinaria y Zootecnia, Universidad Nacional Autónoma de México, Ciudad de Mexico, México; 5 Animal Health Trust, Lanwades Park, Kentford, Newmarket, Suffolk, United Kingdom; 6 Wildlife Conservation Society, Bronx, NY, United States of America; 7 School of Public Health, Li Ka Shing Faculty of Medicine, The University of Hong Kong, Hong Kong Special Administrative Region, China; 8 Laboratory Animal Unit, Li Ka Shing Faculty of Medicine, The University of Hong Kong, Hong Kong Special Administrative Region, China; 9 Weipers Centre Equine Hospital, School of Veterinary Medicine, University of Glasgow, Glasgow, United Kingdom; 10 Boyd Orr Centre for Population and Ecosystem Health, Institute of Biodiversity, Animal Health and Comparative Medicine, College of Medical, Veterinary and Life Sciences, University of Glasgow, Glasgow, United Kingdom; 11 Department of Population Medicine and Diagnostic Science, College of Veterinary Medicine, Cornell University, Ithaca, NY, United States of America; Fred Hutchinson Cancer Research Center, UNITED STATES

## Abstract

Virus ecology and evolution play a central role in disease emergence. However, their relative roles will vary depending on the viruses and ecosystems involved. We combined field studies, phylogenetics and experimental infections to document with unprecedented detail the stages that precede initial outbreaks during viral emergence in nature. Using serological surveys we showed that in the absence of large-scale outbreaks, horses in Mongolia are routinely exposed to and infected by avian influenza viruses (AIVs) circulating among wild birds. Some of those AIVs are genetically related to an avian-origin virus that caused an epizootic in horses in 1989. Experimental infections showed that most AIVs replicate in the equine respiratory tract without causing lesions, explaining the absence of outbreaks of disease. Our results show that AIVs infect horses but do not spread, or they infect and spread but do not cause disease. Thus, the failure of AIVs to evolve greater transmissibility and to cause disease in horses is in this case the main barrier preventing disease emergence.

## Author summary

Viral diseases pose a constant threat to humans and animals. Occasionally, viruses establish in new hosts, sometimes with devastating consequences. While we still do not know what allows a virus to infect and become transmissible in a new population, it is clear that ecology and evolution play an important part in this process. Influenza A viruses (IAVs) constitute the archetypical example of emerging viruses: their main natural reservoir is in wild birds but they have also established in humans, pigs and horses. To better understand how IAVs circulate in nature we sequenced over twenty avian influenza viruses collected from wild birds in Mongolia. We show that these viruses are partially related to a virus that caused an equine influenza epizootic in 1989, that they can infect and replicate in the respiratory tract of the horse without causing any tissue damage, and that -based on serological evidence- horses in Mongolia have been regularly exposed to them over a broad geographical area without causing clinically evident outbreaks. We conclude that equine infections by avian viruses able to replicate in horses are more common than originally thought and that the failure to acquire key genetic changes is in this case the main barrier to disease emergence.

## Introduction

Emerging viral infections pose a constant threat to humans and animals. Viral emergence causing an epidemic of disease in a new host is a rare event, despite the wide diversity of viruses that are known to exist [1] and the constant exposure of hosts to viruses that circulate in other species that share the same habitat. Multiple virus introductions may precede large-scale epidemics, as was seen in the cross-species transmissions of simian immunodeficiency viruses leading to the epidemic spread of the human immunodeficiency virus groups O, N, and P, with only the group M viruses becoming pandemic [2–4]. Transmission of H3N8 equine influenza virus (EIV) to dogs has been reported in Australia [5], the United Kingdom [6] and United States of America [7] but only in the latter did the canine influenza virus (CIV) emerge and become established as a novel pathogen of dogs, in which it has been circulating for over 15 years. In contrast, and despite hundreds of human zoonotic infections being reported for H5N1 avian influenza virus (AIV), this virus has not acquired the ability to transmit among humans [8]. The apparently low proportion of successful cross-species jumps could be explained by the actions of ecological, genetic and immunological barriers barriers. For example, the transmitted pathogens often lack key (but poorly understood) phenotypic features required to undergo efficient transmission, and the structure of the recipient host population may not provide sufficient contacts between infected and susceptible individuals [9]. Viral emergence could also be impaired in the presence of ample ecological opportunities if mutations required to adapt to a new host are difficult to acquire or have a high fitness cost in the donor or recipient host [10]. Indeed, even in the presence of the “right” ecological and evolutionary factors, influenza virus emergence could be impaired by hemagglutinin imprinting, a biological process by which the first IAV infection of an individual confers lifelong cross-protection against severe infection by viruses carrying an HA of the same phylogenetic group [11, 12].

Understanding the ecological and evolutionary factors that favor or impede viral emergence requires the study of spillover events and the so-called “stuttering chains of transmission that lead to the evolution of increased transmissibility” that have been suggested to form the initial stages of viral emergence [13]. To cause outbreaks, spillovers therefore likely require favorable combinations of donor and recipient host ecology leading to exposure; viral-host combinations required for infection; and evolution allowing successful adaptation for increased infection and transmission within the recipient host population. Understanding the relationships between spillover events and emerging outbreaks requires longitudinal monitoring of exposures. While phylogenetics enables to link viral changes with host range shifts, experiments are generally required to understand whether certain mutations that arise during viral emergence cause host adaptations (so-called “gain of function” mutations).

Wild aquatic birds are the main natural reservoirs of most influenza A viruses (IAVs) subtypes. Migratory birds are key for viral spread over long distances, where the viral lineages are often found to be associated with the different flyways, which also favor the generation and maintenance of IAV diversity [14]. H3 AIVs are highly prevalent in wild birds, being found in birds from many different genera across multiple continents [15], and different viruses with the H3N8 glycoprotein combination appear to be particularly prone to transfer into new hosts, having caused outbreaks or epidemics in horses, dogs, pigs, camels and harbor seals [7, 16–19].

The objective of this work was to examine interspecies transmission of H3 AIVs to horses in Mongolia, and to determine the relative contribution of ecological and evolutionary processes that precede, and which might lead to viral emergence. Horses are a suitable species to study the biological processes that underpin the transfer, adaptation and establishment of IAVs in mammals: they have supported at least four major IAV epidemics, including a virus of unknown serotype around 1872, an H7N7 that emerged in Czechoslovakia in 1956 and died out in the 1970s [20], and two phylogenetically distinct H3N8 viruses. The first one emerged in 1963, became endemic in horse populations and still circulates in most countries [16]. The second one caused a large epizootic in Jilin, China, in 1989 and circulated in horses for only a couple of years before dying out [21, 22]. While the direct ancestors of the equine viruses have not been identified, phylogenetic studies of the three known viruses indicate that they likely arose from direct transfer from avian hosts [21, 23, 24].

Mongolia possesses favorable ecological features for influenza emergence in horses: it contains a large horse population (~3.3 million) [25] and sizable numbers of migratory waterfowl and shorebirds [26]. The country lies at the intersection of two major migratory flyways, hosting migrants from as far as the Indian Subcontinent and Australasia that arrive in the spring to breed or pass through to nesting areas further north. From mid-summer large congregations of Anseriformes gather in Mongolia to molt, before returning south in the autumn. Finally, H3N8 EIVs and AIVs co-circulate in Mongolia [27, 28] opening up the possibility of reassortment if co-infections occur.

Here we examined through phylogenetic, serological and experimental approaches the ecological and evolutionary factors mediating viral emergence. To this end, we combined surveillance and sequencing of viruses from wild birds to allow phylogenetic analysis of a large number of influenza virus genomes, and also performed prospective and retrospective seroepidemiological analysis of horses from Mongolia (and the United Kingdom as a control population) to identify ecological factors associated with AIV interspecies transmission. We isolated and sequenced EIVs from Mongolian horses to determine whether AIV-like viruses were present. We also carried out experimental infections of equine tracheal explants with Mongolian AIVs to determine whether the viruses were able to infect and replicate in the respiratory tract of the horse.

## Results

### AIVs circulating among wild birds in Mongolia are closely related to an avian-like equine influenza virus that caused a large epidemic in horses in Asia between 1989–1990

**Fig 1:**
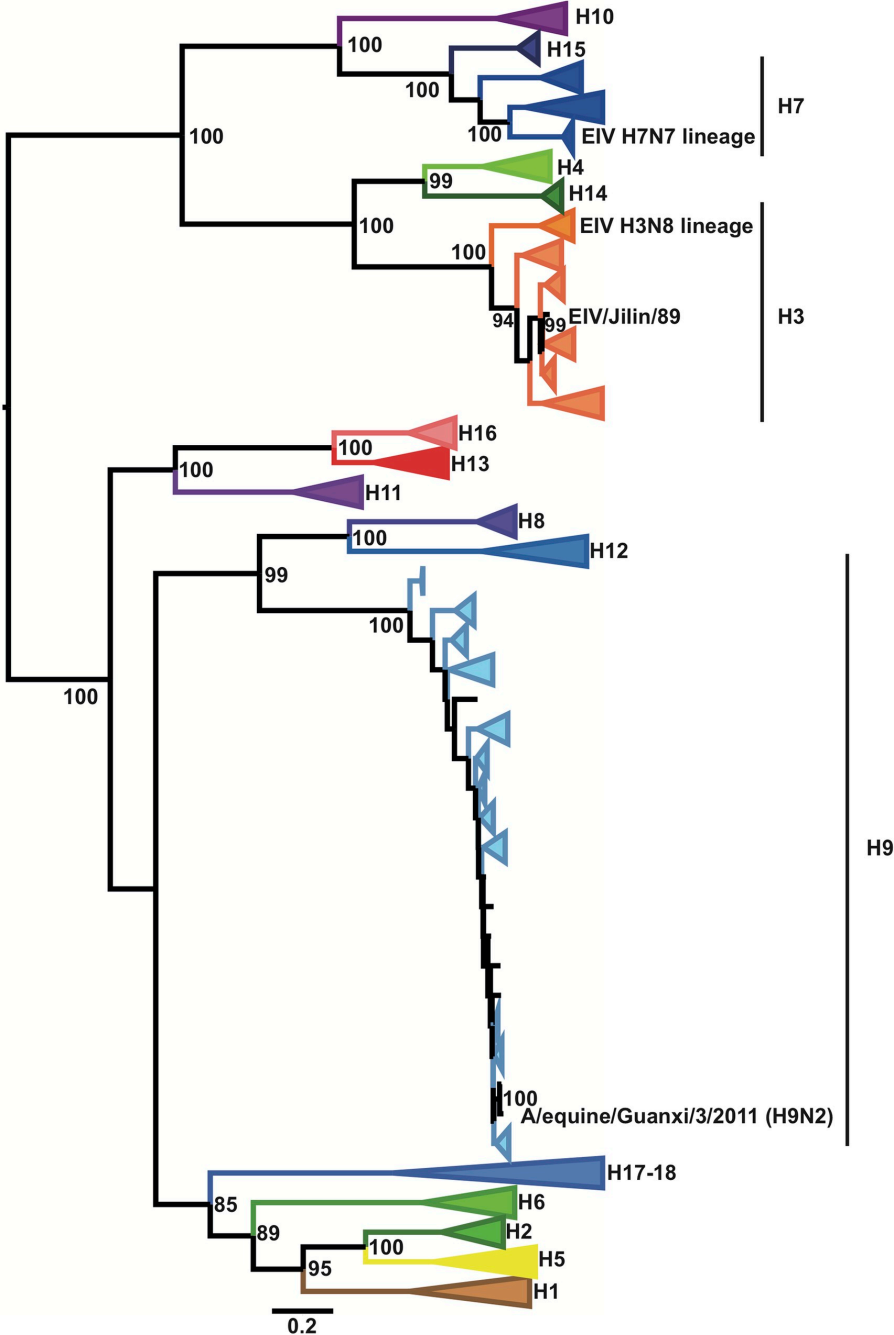
Phylogenetic relationship of AIVs isolated from wild birds in Mongolia between 2009 and 2011. Maximum likelihood tree using a sequence dataset comprising 860 HA IAV sequences representing 21,277 IAV genomes. Colored triangles represent lineages that correspond to individual HA subtypes as indicated. The H3N8 and H7N7 EIV lineages are indicated with green and red circles, respectively. Bootstrap values are indicated next to relevant nodes. Scale bar represents the number of substitutions per site.

**Fig 2:**
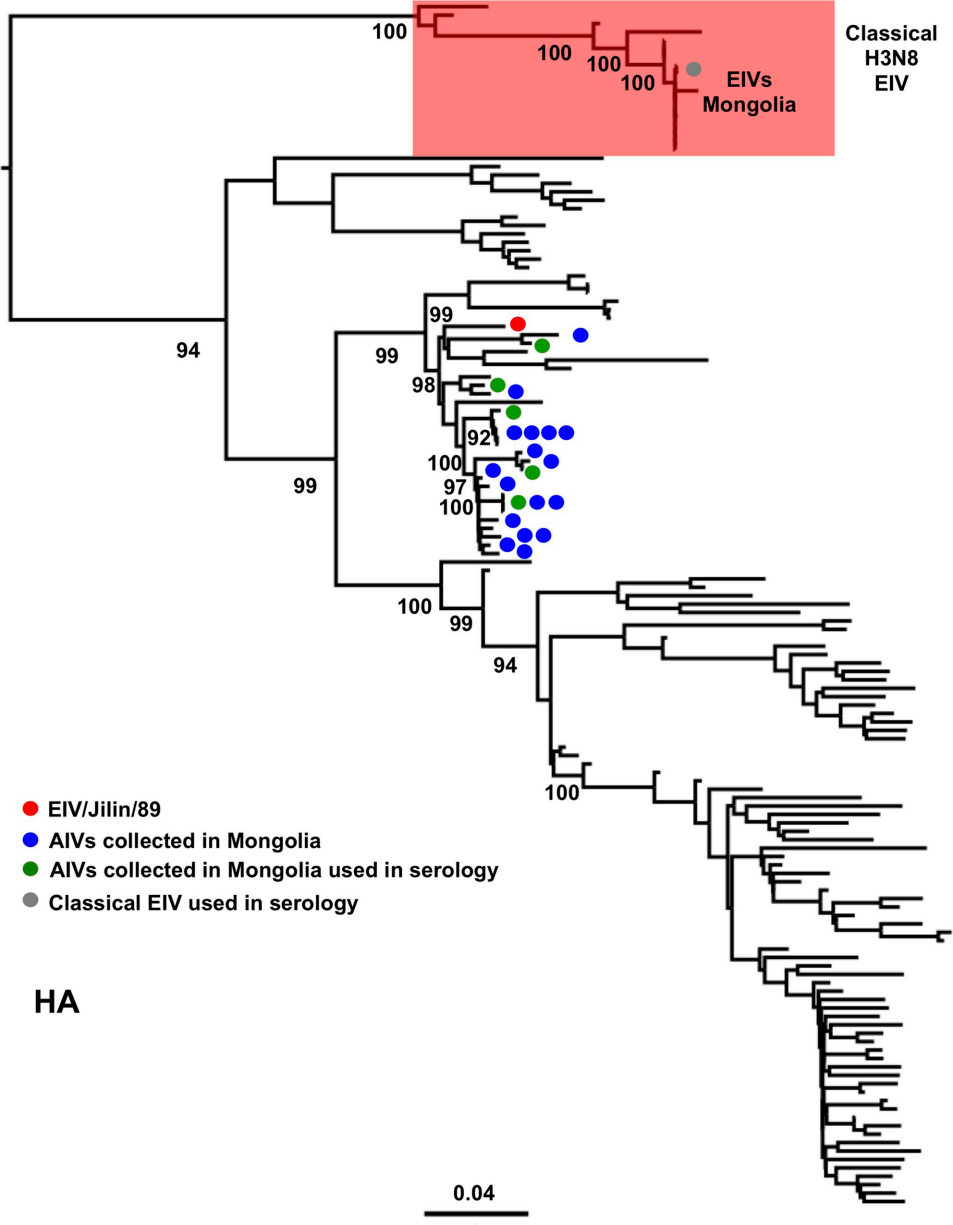
Phylogenetic relationship of viruses within the H3 lineage. EIV/Jilin/89 is marked in the phylogeny with a red circle, AIVs isolated in Mongolia (AIVs/2009-11) are indicated with blue and green circles (the latter represent the isolates used in HA assays) and the currently circulating H3N8 EIV lineage (“Classical EIV”) is indicated with a red box. The scale bar represents the number of substitutions per site. Bootstrap values are indicated next to the relevant nodes.

**Fig 3:**
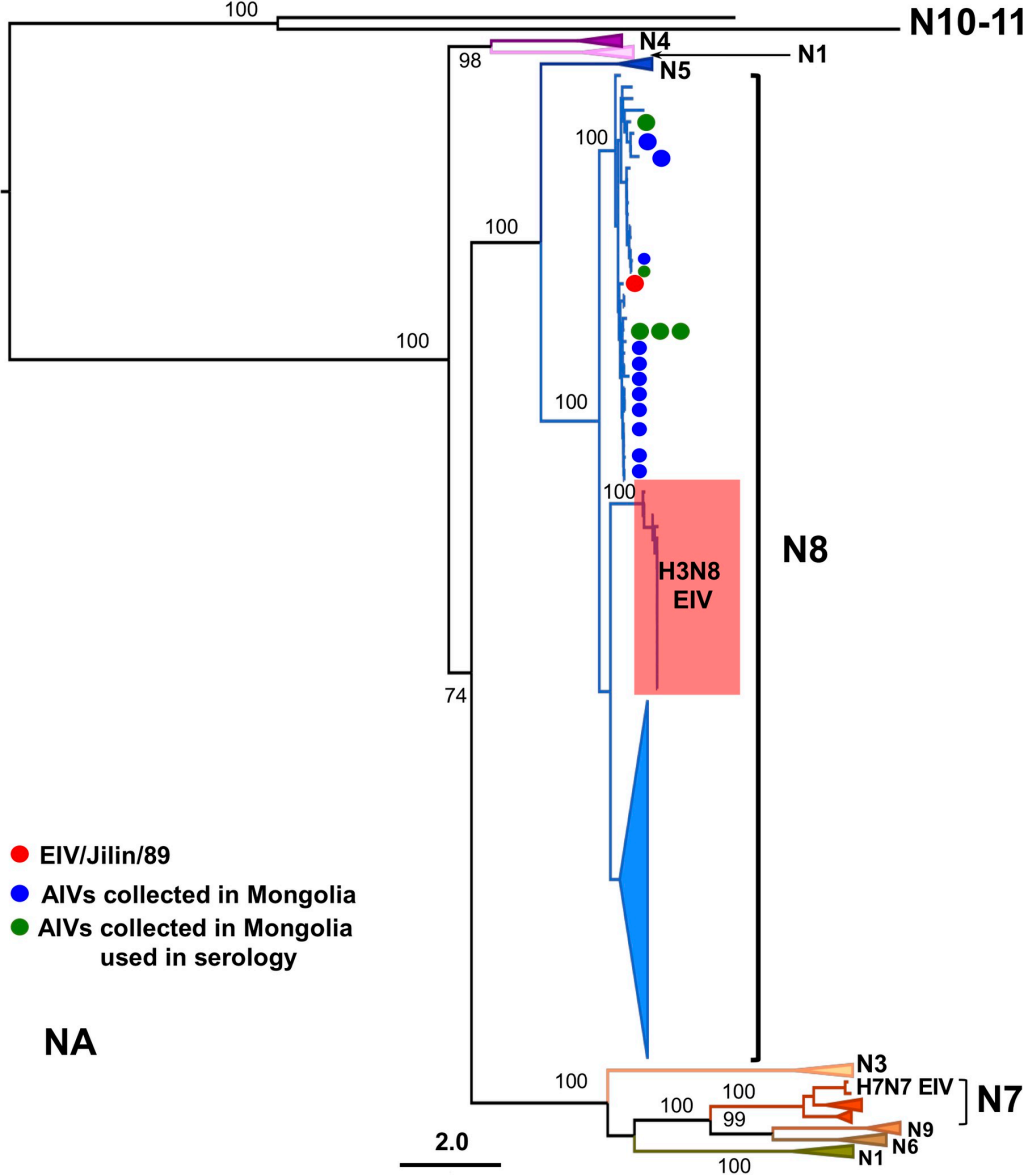
Phylogenetic relationship of the NA genes of AIVs isolated from wild birds in Mongolia between 2009 and 2011. Maximum likelihood tree using a sequence dataset comprising 859 IAV sequences representing 21,276 IAV genomes. Colored triangles represent lineages that correspond to individual NA subtypes as indicated. AIVs isolated in Mongolia (AIVs/2009-11) are indicated with blue and green circles (the latter represent the isolates used in HA assays) and the currently circulating H3N8 EIV lineage (“Classical EIV”) is indicated with a red box. The scale bar represents the number of substitutions per site. Bootstrap values are indicated next to relevant nodes.

**Fig 4:**
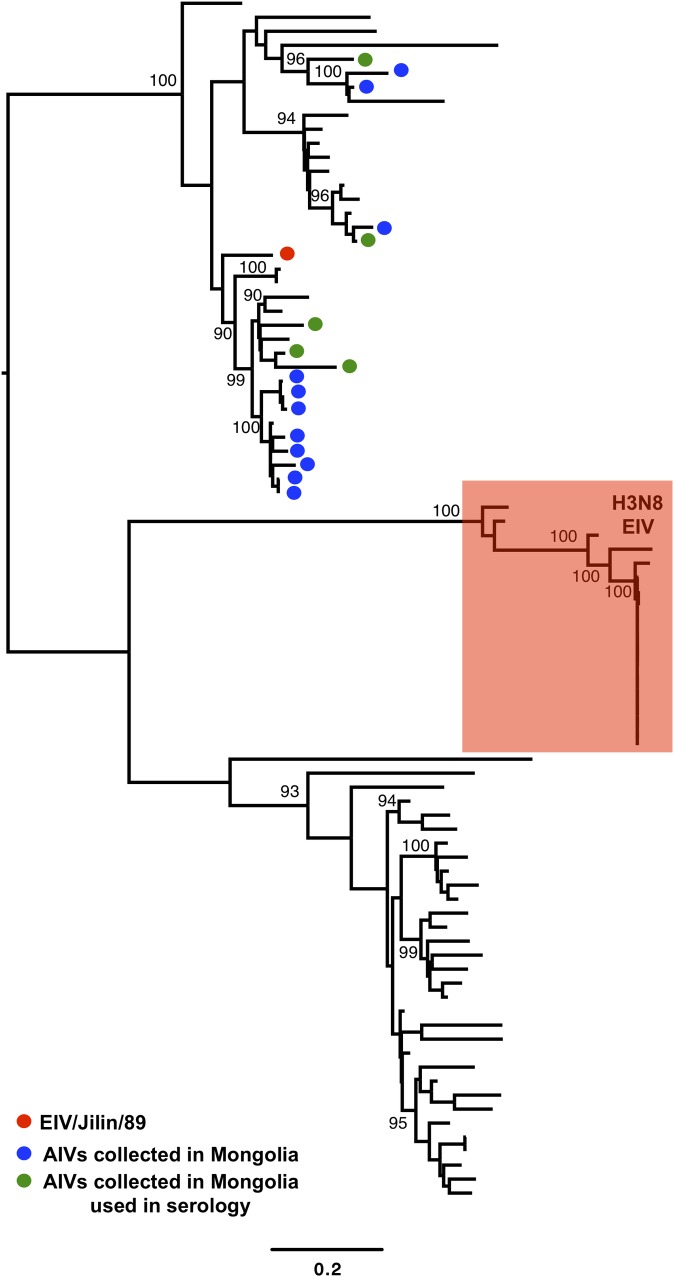
Phylogenetic relationship of viruses within the N8 lineage. EIV/Jilin/89 is marked in the phylogeny with a red circle, AIVs isolated in Mongolia (AIVs/2009-11) are indicated with blue and green circles (the latter represent the isolates used in HA assays) and the currently circulating H3N8 EIV lineage (“Classical EIV”) is indicated with a red box. The scale bar represents the number of substitutions per site. Bootstrap values are indicated next to the relevant nodes.

Longitudinal surveillance studies carried out in wild birds in Mongolia between 2008 and 2012 indicate that H3 AIVs are highly prevalent [29]. As H3N8 AIVs have emerged in horses in two independent occasions [16, 21], we isolated and sequenced the genomes of 22 H3 AIVs (including 17 H3N8 viruses) collected from wild birds in Mongolia between 2009 and 2011 to determine whether they were phylogenetically related to EIVs (S1 Table). We inferred phylogenetic trees for each genomic segment from multiple sequence alignments that represented ~21,300 IAV genomes obtained from publicly available databases. Interestingly, the haemagglutinin (HA) genes of all Mongolian AIV isolates were closely related to A/equine/Jilin/89 (referred to as EIV/Jilin/89, Figs 1 and 2), an avian-origin influenza virus that caused a large epidemic of respiratory disease in horses in 1989 and in the early 1990s [21]. Phylogenetic analysis of individual gene segments showed that the neuraminidase (NA, Figs 3 and 4) and matrix protein (MP, S1 Fig) genes of 11 of the 22 Mongolian AIVs clustered together with high support with EIV/Jilin/89. Further, the polymerase acidic gene (PA) of AIV/963 was also very closely related with that of EIV/Jilin/89 (S2 Fig), while the non-structural gene (NS) of 19 of the AIVs showed a relatively close phylogenetic relationship with EIV/Jilin/89 (S3 Fig). In contrast, the polymerase basic 2 (PB2), polymerase basic 1 (PB1) and nucleoprotein (NP) genes (S4–S6 Figs) of all AIVs sequenced exhibited a distant phylogenetic relationship with EIV/Jilin/89. These results indicate that AIVs bearing several genes closely related to EIV/Jilin/89 were still circulating in wild birds in Mongolia between 2009 and 2011.

### Identification of avian-to-horse adaptive mutations

To identify amino acid mutations in AIVs that might play a role in horse adaptation we used a site-wise non-homogeneous phylogenetic model developed by Tamuri et al. [30], which takes into account the phylogenetic relationships between the viruses. This method identified 17 mutations (Table 1) distributed in six of the eight IAV genomic segments. Notably, 15 of those amino acids were present in both the AIVs we sampled in Mongolia and an H3N8 equine virus (either Jilin/89 or what we called the classical EIV lineage). Further, two amino acid changes (D701N in PB2 [31] and S345N in NP [32]) have been shown to mediate mammalian adaptation of avian viruses. These results suggest that AIVs circulating in Mongolia already harbor some, but not all the mutations that might be required for successful interspecies transmission and emergence in horses.

**Table 1:** Putative host-differentiating sites between EIVs and AIVs.

		Amino acid frequencies		
	Amino acid residue	Classical EIV (%)	EIV/Jilin/89	MN AIVs (%)
PB2	377	A (78), E (4), T (14), V (3)	A	A (100)
	380	G (1), K (3), R (92), S (3)	R	R (100)
	586	R (100)	K	K (100)
	701	N (100)	N	D (100)
PB1	157	S (100)	A	A (100)
	429	R (98), 2 X	K	K (100)
HA	209 (193^1^)	E (2) K (98)	E	21N
NP	41	V (100)	I	I (100)
	117	I (1), M (99)	R	R (100)
	245	G (100)	S	S (100)
	345	N (100)	S	S (100)
	351	K (100)	R	R (100)
	384	56 R, 37 K	K	R (100)
NA	201	I (42), T (1), V (57)	I	16 V
NS	139	A (1), E (99)	D	D (100)
	171	N (100)	T	19 T, 3 D
	214	F (100)	L	22L

Amino acid sites were identified using a site-wise non-homogeneous phylogenetic model [30]. For each segment, a codon alignment was generated and sequences were assigned to avian and equine groups. The topology of the phylogenetic tree for each segment was reconstructed based on codon alignments and branch lengths were re-optimised based on corresponding amino acid data. The sitewise non-homogeneous phylogenetic model [30] was then used to identify the amino acid sites with changes in selective constraints following the host shift from avian to equine species. The table shows sites with false detection rates (FDR) lower than 0.1.

^1^ Refers to the amino acid position in the unaligned protein using the H3 (A/AICHI/2/68) sequence numbering scheme.

### Horses in Mongolia are continuously exposed to avian and equine influenza viruses

**Fig 5:**
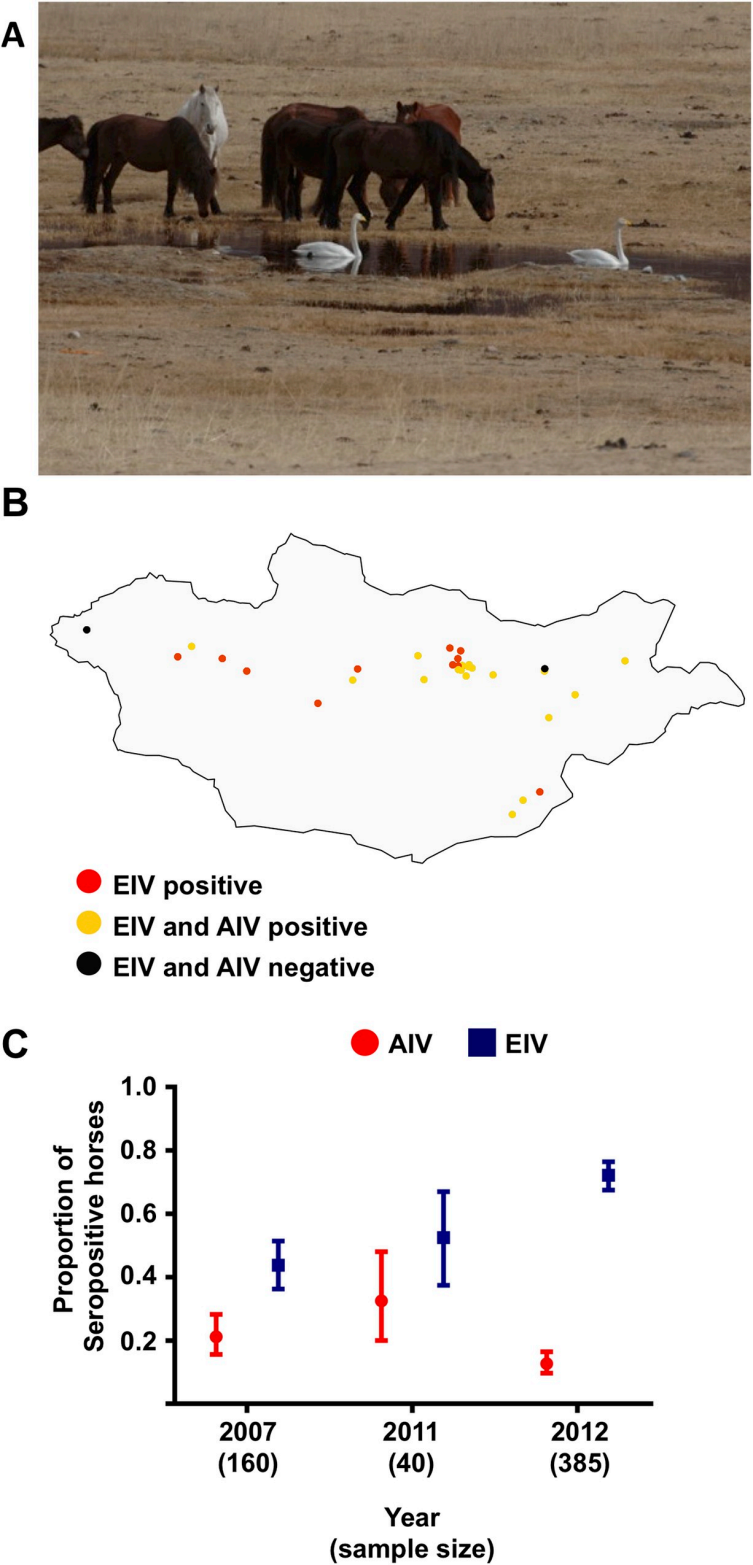
A. Photograph showing the interface between horses and wild birds in Mongolia. B. Geographical distribution of sampling sites. Each circle represents a site where multiple horses were tested. Sites where horses were seropositive to EIV only are shown as red circles. Sites where horses were seropositive to both AIV and EIV are shown as yellow circles. Sites were horses were seronegative to both AIV and EIV are shown as black circles. C. Serological detection of EIV and AIV in horses in Mongolia. The graph shows the proportion and Agresti-Coull confidence interval of seropositive horses.

Horses and wild birds in many areas of Mongolia share the same habitat and exhibit high contact rates, particularly during the summer (Fig 5A), so we examined whether horses had been exposed to and infected by AIVs. Hemagglutination inhibition (HI) assays were conducted on sera that had been collected from horses during outbreaks of equine influenza that took place in 2007–8 and 2011, as well as on sera collected from healthy horses during routine surveillance in July 2012. Samples were collected from various geographical locations across Mongolia (Fig 5B). We used five H3N8 AIVs as antigens in the HI assays (AIV/881, AIV/963, AIV/2076, AIV/2106 and AIV/2271, marked as green circles in Fig 2).

As EIV is endemic in Mongolia and all the AIVs that we collected in Mongolia are of the same HA subtype (H3) we sought to rule out any cross reactivity between them to avoid false positive results. We thus determined if there was cross-reactivity between H3N8 EIV and the AIVs under study. To this end, we carried out HI assays using the aforementioned avian viruses together with an H3N8 EIV, against a set of antisera from convalescent horses that were known to have been infected with H3N8 EIVs in the US and which were unlikely to have been exposed to AIVs circulating in Mongolia. Table 2 shows that there was no cross-reactivity between AIVs and EIVs.

**Table 2:** Serological relationships among H3N8 equine influenza viruses and avian influenza viruses isolated from wild birds in Mongolia.

		HI titers to the following equine antisera							
		Ohio/03	Ky/2/07	Ky/4/07	Ky/7/07	Ky/91	Ky/99	Ky/5/02	Naive
**Viruses**	**Ohio/03**	640	640	1280	320	160	640	640	Neg.
	**Ky/5/02**	320	640	640	640	80	1280	1280	Neg
	**Ky/4//07**	640	640	640	640	160	640	320	Neg
	**Ky/7/07**	320	320	320	320	40	320	320	Neg
	**Ky/91**	160	320	320	160	80	320	320	Neg
	**Ky/95**	80	160	160	160	40	320	160	Neg
	**Ky/99**	80	160	160	160	20	320	160	Neg.
	**AIV/881**	Neg	Neg	Neg	Neg	Neg	Neg	Neg	Neg
	**AIV/963**	Neg	Neg	Neg	Neg	Neg	Neg	Neg	Neg
	**AIV/2076**	Neg	Neg	Neg	Neg	Neg	Neg	Neg	Neg
	**AIV/2106**	Neg	Neg	Neg	Neg	Neg	Neg	Neg	Neg
	**AIV/2271**	Neg	Neg	Neg	Neg	Neg	Neg	Neg	Neg

Hemagglutination inhibition (HI) titers are expressed as the reciprocal of the highest antiserum dilution inhibiting 4 hemagglutinating units (HU) of virus. Equine influenza viruses are underlined.

**Fig 6:**
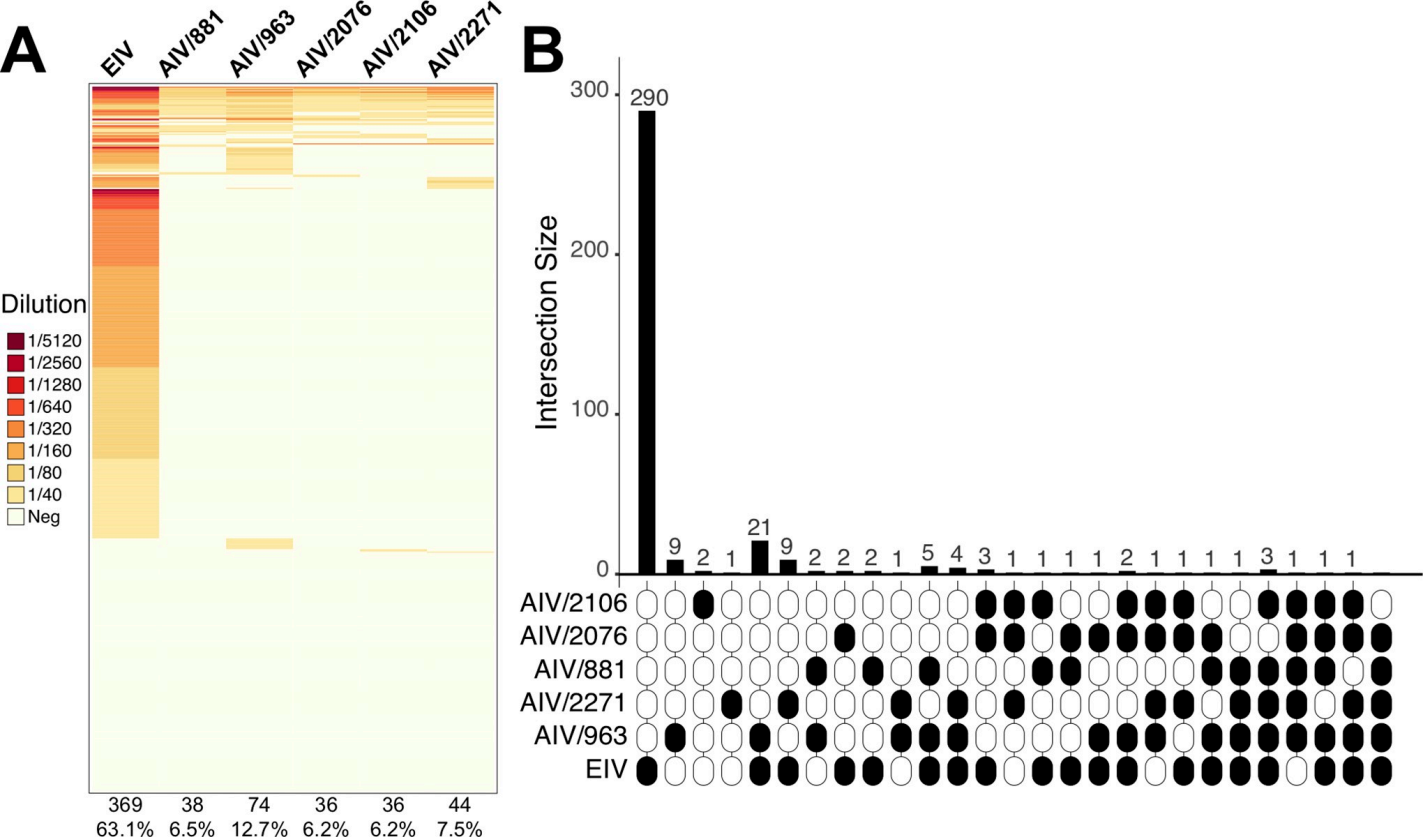
Individual serological results for AIVs and EIV. A. Heat map showing the HI value for all viruses used in serological assays. Each row represents an individual horse. Each column shows the virus tested in bold, the number of seropositive samples, and the percentage of seropositive samples within brackets. HI values are color-coded according to the key, with darker colors representing higher levels of seropositivity. B. UpSet plot showing the number of seropositive horses to one or more viruses. Black and white ellipses represent seropositive and seronegative samples, respectively. Bars indicate the number of seropositive horses to each virus combination.

In the HAI tests we observed a relatively high proportion of AIV-seropositive horses each year (Fig 5C). The lowest proportion was observed in 2012 (~0.13, Agresti-Coull interval = 0.1–0.16), while the highest was observed in 2011 (0.32, Agresti-Coull interval = 0.2–0.48). Of note, of the 97 horses that were seropositive to AIVs, 18 were negative to EIV indicating exposure to avian but not equine influenza viruses. Age information was only obtained from horses sampled in 2012. From this group, 41 horses were seropositive for both AIVs and EIV and the median age of those animals was three years, indicating that exposure to both viruses occurred over a relatively short time period. We observed a significantly higher proportion of EIV seropositive animals in all years compared to AIV (p = 0.05, two-sample z-test), consistent with EIV being endemic in Mongolia. It should be noted that an EIV vaccine is available. However, as it is very rarely used, we assumed that our serology results reflect natural infections. Results on individual testing for each animal are available in Fig 6 and S2 Table.

Since some of the sera that were positive to AIVs were collected during equine influenza outbreak investigations in 2007 and 2011, we performed virus isolation on nasal swabs collected during those outbreaks. We sequenced their complete genomes and observed that each of their genomic segments belonged exclusively to the classical H3N8 EIV lineage (Fig 2). No AIV sequences were obtained from sampled horses.

To confirm the HAI results, all serum samples that were positive for AIV by HI were further tested using microneutralization assays, which showed a high level of consistency (91.7%, S1 Appendix).

As contact or close association between donor and recipient host species is a prerequisite for cross-species infections, we used as a control the same antigens described above to perform HAI assays on 60 serum samples collected in 2012 from healthy Welsh mountain ponies in Shropshire, United Kingdom. These horses are maintained in similar conditions to those in Mongolia (i.e. non-stabled, free-roaming). As expected, all samples were negative to all viruses (S2 Table).

These results indicate that ecological features in Mongolia facilitate exposure of horses to AIVs circulating in wild birds on a regular basis and over broad geographical areas.

### H3N8 AIVs can infect and replicate in the respiratory tract of the horse

Viral emergence requires efficient replication of the virus in cells and tissues of the novel host. This is a highly specific process that includes multiple steps including receptor binding, virus entry, gene expression, genome replication, virus assembly, and release of infectious virus that transmits to new individuals of that host species. In alternative hosts, species barriers impede infection by novel viruses, presumably by blocking one or more of those steps. To test whether AIVs isolated from wild birds in Mongolia could infect and replicate in the respiratory tract of the horse, we inoculated horse tracheal explants with the same AIVs used in our serological survey, as well as with a classical H3N8 EIV isolate (A/equine/South Africa/2003). We monitored infected explants for five days. This timeframe was used because in natural infection horses start showing symptoms and shedding virus as early as 24 hours post-infection [33] and histopathological lesions are evident at 48 hours post-infection [34].

**Fig 7:**
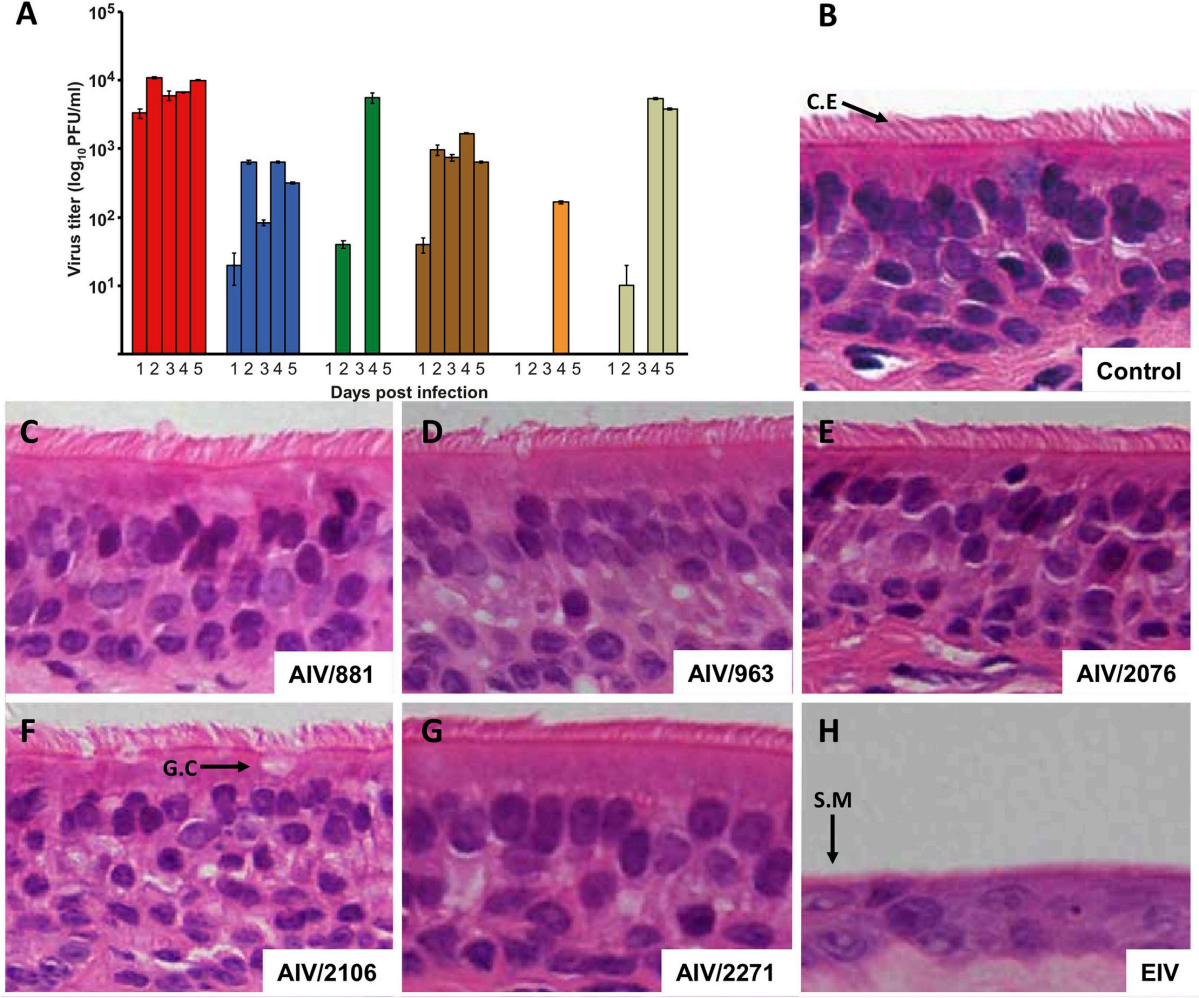
Infection phenotype of equine and avian influenza viruses in horse trachea. A. Growth kinetics of the indicated viruses in horse tracheal explants. Vertical bars represent average titers from three experiments. Dotted line indicates the titer of the inoculum used to infect the explants. B. Light micrographs of an H/E stained section of an uninfected horse tracheal explant. C to H. Light micrographs of H/E stained sections showing explants infected with EIV and indicated AIVs. Ciliated epithelium (C.E), goblet cell (G.C) and squamous metaplasia (S.M) are indicated with arrows. Images magnification: 400X.

With the exception of AIV/2106 all H3N8 AIVs tested were able to infect and replicate in the horse trachea, but they replicated to lower levels than EIV (Fig 7A). A clear difference between AIVs and EIV was observed when histopathological changes were assessed. EIV infection (Fig 7H) caused evident lesions including loss of ciliated epithelium, squamous metaplasia, necrosis, apoptosis and reduction of goblet cells similar to those observed *in vivo* [34]. In contrast, explants infected with AIVs did not exhibit any evident microscopic lesions (Fig 7C to 7G). Overall, these results suggest that some H3N8 AIVs circulating in wild birds in Central Asia have the necessary attributes to successfully infect and replicate in the respiratory tract of the horse, but that they lack the properties necessary to cause tissue damage in the horse trachea.

## Discussion

The emergence of epidemic viruses is a rare event that requires both exposure of the new host, as well as the correct properties of the virus to allow efficient onward transmission among individuals of the new host species. As a result, viral emergence can be influenced across different scales: from molecular interactions between virus and host proteins or glycan receptors, to ecosystem landscapes that determine the geographical distribution and contact rates between donor and recipient species [35]. Multidisciplinary studies that combine field, phylogenetic and experimental approaches are essential to understand how viruses infect and spread in new host populations.

The transfer of IAVs to new hosts is relatively rare and appears to occur on only a limited number of mammalian hosts, including humans, swine, seals, horses and dogs, as well as poultry. In many other cases influenza viruses are seen to spill over to cause individual infections in a new host with limited capacity for transmission in the new host–as is seen for H5N1, H7N9 and H10N8 IAVs to name a few [8, 36, 37].

Here we documented with great detail the frequent exposure of horses to AIVs that are genetically close to EIV/Jilin/89. This is consistent with repeated spillover infections by viruses near a genotype that caused an epidemic in the past and thus had an R_0_>1. As horses are clearly permissive hosts for some IAVs, their study provides opportunities to ask fundamental questions about viral emergence that can lead to new insights into the processes that are associated with virus spillovers and emergence.

A proportion of the circulating AIVs in birds carry some mutations that have been shown to be associated with mammalian adaptation. While this observation implies that some AIVs are closer to an equine genotype, adaptation might require epistatic interactions that could be specific (and different) for EIV/Jilin and Classical EIV. In addition, some AIVs efficiently infected the equine respiratory tract in culture, suggesting that they can successfully infect horses–which is supported by the finding of antibodies against those viruses in 10–30% of the horses sampled in Mongolia.

Why have large outbreaks of disease caused by avian-origin influenza viruses not occurred in horses in Central Asia over the last 25 years? Different scenarios could explain this situation and the data presented here does not favor any particular one. In the first scenario, it is likely that AIVs circulating in wild birds are not genetically adapted to transmit among horses. This is supported by the presence of some but not all of the mutations that are shared between the two H3N8 epizootic viruses (Table 1). In addition, all AIVs examined harbored three genomic segments (PB2, PB1 and NP) that were distantly related to EIV/Jilin/89, and in other cases of viral emergence in new hosts, mutations in those segments control virus replication and transmission [32, 38, 39]. While our results suggest that there is an "emergence ready" genotype (EIV/Jilin/89) in the genetic neighborhood of the AIV strains circulating in Mongolia, we do not know how many mutations will be required for a new EIV to emerge. Experiments that are beyond the scope of this work -such as site directed mutagenesis or serial passaging of AIVs in equine cells- will be required to answer this question.

A second possible scenario is that AIVs are being transmitted from horse to horse (albeit not very efficiently) and the circulating viruses are undetected. This is supported by the lower seroprevalence of AIVs when compared to EIV and by the absence of lesions observed in our experimental infections, which suggest that infected animals might not display clinical disease. If this were the case, we may be observing low levels of transmission that are the preceding stages of influenza emergence and one of the viruses may evolve to become more transmissible in horses by gaining adaptive mutations [40, 41]. Experimental transmission experiments could help to investigate this hypothesis.

A third possibility that should be considered is cross-protective immunity resulting from hemagglutinin imprinting [11, 12]. Since 1974 there have been five EIV epizootics documented in Mongolia: 1974–1975, 1983–1984, 1993–1994, 2007–2008, and 2011 [28]. As vaccination coverage in the country is seemingly very low [42], it can be reasonably assumed that most seropositive horses have acquired EIV immunity by natural infection, which is known to induce a potent immunological response. Since all AIVs studied here possess the same subtype as EIV (H3), the high seroprevalence of the latter could provide enough pre-existing immunity against H3 AIVs, thus acting as an effective barrier against AIV emergence in horses. While this seems counterintuitive as we showed that EIV does not cross react with H3 AIVs in HI assays, it has been shown that even viruses possessing different HA subtypes can elicit cross-protective immunity [12].

How could avian-origin influenza viruses emerge in horses? They may evolve higher transmissibility through acquisition of point mutations. The likelihood of viral emergence through this process will depend on the number of mutations that separate the viral replication and transmission fitness peaks in the donor and the recipient species [43]. Our results suggest that the evolutionary landscape that H3N8 AIVs would have to traverse to emerge in horses might not be particularly steep as they can already infect and replicate in the respiratory tract of the horse (Fig 5), as has happened on at least two occasions in the past. Since there is continuous spillover occurring and the IAVs that we sequenced already harbor putative adaptive mutations this seems a likely scenario (Table 1).

Alternatively, or in addition, the IAVs may acquire the “right” genomic constellation by reassortment either in birds or in horses. While reassortment in birds frequently occurs, it seems unlikely to give rise to equine infecting viruses given the seemingly random distribution of genome constellations present in wild birds [14] and the likely selective pressure for avian -rather than equine- adapting viruses. Emergence of novel EIVs via reassortment in horses seems more probable, particularly since we observed that horses as young as two years old are being infected by both avian and equine viruses, opening up the possibility of coinfections. Also, selection pressures for equine-adapted viruses are more likely to occur in horses. Future experiments using AIV/EIV reassortant viruses generated by reverse genetics will address this issue.

It should be noted that we sequenced 13 viruses isolated from horses during outbreaks of equine influenza and did not detect any avian-like viruses, nor any AIV-EIV reassortant virus. However, our dataset shows that an H9N2 avian-like influenza virus was isolated from horses in 2011 in Guanxi, China (Fig 1). Including this event, there have been four reported independent introductions of AIV in horses, three of which led to EIV emergence (i.e. H7N7 in 1956 [Prague], H3N8 in 1963 [Miami], and H3N8 in 1989 [Jilin]). In addition, a previous study reported an outbreak of H5N1 influenza virus in donkeys living in close proximity with H5N1-infected poultry in Egypt in 2009 [44]. As horses and donkeys are closely related, it is feasible to think that H5N1 AIVs might infect horses. Animal management measures should be considered in H5 endemic areas where horses and poultry are in close contact.

In sum, our results advance our understanding of what it takes to allow IAVs to emerge successfully in a mammalian host. We identified a geographical “hotspot” [45] for viral cross-switching where future large-scale disease outbreaks appear to depend on evolutionary factors. Surveillance efforts should be directed to monitor for the presence of horse adaptive mutations in viruses derived from birds and on additional serological surveys to determine exposure levels in horses. Our results also show that horses are susceptible to infection by some AIVs, but that they likely display effective species barriers that prevent the establishment of novel IAVs. Future studies aiming at understanding those blocks to cross-species transmission using avian and equine influenza viruses will provide insight on the mechanisms and determinants that underpin influenza emergence in mammals.

## Materials and methods

### Sampling and virus isolation

Avian influenza viruses were obtained during surveillance studies carried out in Mongolia [46]. Sampling methods and virus isolation techniques have been previously described [46]. Equine influenza viruses were isolated from horses during routine surveillance in Mongolia and during outbreak investigations. Virus isolation in 9–12 day old embryonated chicken eggs was performed by the Mongolian State Central Veterinary Laboratory (SCVL) following WHO guidelines for the isolation of animal influenza viruses [47].

### Genome sequencing and assembly

Viral RNA was extracted using the QIAmp viral RNA minikit (Qiagen) following the manufacturers’ instructions. Reverse transcription followed by PCR amplification were performed as previously described [48] and amplicons were prepared and sequenced using the Illumina MiSeq platform. Reads were quality controlled and trimmed using FAstQC and Trim Galore. The cleaned reads were then *de-novo* assembled using Velvet [49]. The assembled contigs where used to identify the most appropriate reference to use for each segment using BLAST against the influenza database on GenBank and the retrieved references were used for a reference based assembly using Stampy [50]. All AIV genomes sequenced were deposited in the NCBI taxonomy database (https://www.ncbi.nlm.nih.gov/taxonomy) and the taxonomy ID numbers are included in S1 Table.

### Phylogenetic analysis

We downloaded 21,242 completed influenza A virus genomes from the NCBI Influenza Virus Resource Database. For each segment, a codon alignment was generated by using MUSCLE [51] and Pal2nal [52], and manually edited using SeaView [53]. All eight segments were concatenated into full genome sequences. We used CD-HIT [54] to cluster the full genome alignment, and this resulted in a reduction of the original dataset to 825 clusters (sequences that shared ≥97% identity were grouped together). Phylogenetic trees were inferred using one representative sequence from each of the 825 clusters generated by CD-HIT, together with the genomes of the 22 influenza viruses collected from wild birds (S1 Table) and 13 influenza viruses isolated from horses in Mongolia (S3 Table). The final data set comprised 860 sequences for each segment. Codon alignments were then realigned for each segment using MUSCLE. We used RAxML [55] to infer the phylogenetic tree for each segment using the GTRCAT model and 1000 bootstrap replicates.

### Expanded IAV data set

After the maximum likelihood (ML) trees derived from the multiple IAV data set were inferred, we selected a subset of sequences comprising the subtrees containing all H3N8 EIVs and closely related lineages. This data set was expanded to its original number of taxa (before using CD-HIT) with varying number of taxa: 329 for PB2, 98 for PB1, 202 for PA, 257 for HA, 113 for NP, 276 for NA, 981 for M1 and 148 for NS1. Sequences of EIV/Jilin/89 were removed from PB2, PB1, PA, NP, M1 and NS1, as this virus is closely related to avian influenza instead of equine influenza according to the phylogeny. All sequences from other mammals were removed. This left a sequence dataset for each segment with an outgroup of avian sequences and a separate monophyletic equine H3N8 clade corresponding to the host shift event of 1963.

### Selective constraints

For each segment, a codon alignment was generated by using MUSCLE. We assigned all sequences to avian and equine groups according to their host. The topology of the phylogenetic tree for each segment was reconstructed based on codon alignments using RAxML with GTR+G model. Branch lengths were re-optimised based on corresponding amino acid data. This step was achieved by using RAxML with the FLU+G model. The sitewise non-homogeneous phylogenetic model of Tamuri et al [30] was used to identify the amino acid sites with changes in selective constraints following the host shift from avian to equine species. This was performed on each segment based on the re-optimised phylogenetic tree and the corresponding amino acid alignment. Only reported sites with false detection rates (FDR) lower than 0.1 were accepted.

### Equine sera

Serological assays were performed on 385 serum samples collected during routine surveillance carried out in July 2012 by the State Central Veterinary Laboratory of Mongolia. Additionally, archived serum samples collected during outbreak investigations that took place in 2007–08 (n = 160) and 2011 (n = 40) were available for this study. Serum samples collected by the Animal Health Trust in 2012 for unrelated studies from 60 Welsh Mountain ponies bred and kept in Shropshire, UK were also tested.

To determine if there was serological cross-reactivity between AIVs and H3N8 EIVs we used a panel of archived equine sera collected from convalescent horses infected with known EIVs. Sera were against the following viruses: A/Equine/Ohio/2003, A/Equine/Kentucky/2/2007, A/Equine/Kentucky/1/91, A/Equine/Kentucky/4/2007, A/Equine/Kentucky/7/2007, A/Equine/Kentucky/99, and A/Equine/Kentucky/5/2002).

### Viruses

For haemagglutination inhibition assays, sera were tested against A/ruddy shelduck/Mongolia/881V/2009, A/ruddy shelduck/Mongolia/963V/2009, A/common shelduck/Mongolia/2076/2011, A/common shelduck/Mongolia/2106/2011, A/common teal/Mongolia/2271/2011 and A/equine/Ohio/2003. To test for serological cross reactivity between AIVs and EIVs we used A/equine/Kentucky/2/2007, A/equine/Kentucky/1/91, A/equine/Kentucky/4/2007, A/equine/Kentucky/7/2007, A/equine/Kentucky/99, and A/equine/Kentucky/5/2002. For experimental infections of equine tracheal explants we used A/equine/South Africa/2003 and the same AIVs enumerated above.

### Serology

Horse antisera were treated with receptor destroying enzyme (RDE, SEIKEN) and heat inactivated. HI assays were carried out using chicken red blood cells (RBCs) following standard procedures [47]. Sera that inhibited hemagglutination at a dilution ≥ 1:40 were considered positive. Horse antisera that tested positive to AIVs were further tested using microneutralisation assays as described in [47]. We used the Prevalence package in R (version 3.2.2) to calculate seroprevalences including confidence intervals using the Agresti-Coull interval with 0.95 confidence. Maps were generated in R using the Maps package to plot sampling sites. The serology heatmap and UpSet plot were generated in R using the ggplot2 and UpSetR packages, respectively. Data used in the heat map was manually ordered based on serological profiles, and HI values transformed to dilution factors. For the UpSet plot, data was converted to a binary matrix (i.e. positive or negative).

### Explant preparation, infection, processing and virus titration

Horse tracheas were aseptically collected upon euthanasia from healthy Welsh Mountain ponies and prepared as previously described [56–58]. Animal work was approved by the Ethics Committee of the School of Veterinary Medicine of the University of Glasgow (ethics approval R25A/12). As no regulated procedures were carried out on animals a Home Office license was not required. Explants were infected with 200 plaque-forming units (PFU) of each virus directly on the epithelium layer. Culture medium (5 μl) was deposited on the surface of mock-infected explants. For histological analysis explants were fixed in 10% (v/v) buffered formalin and paraffin embedded. Paraffin sections were subject to Haematoxylin and Eosin staining and histological images were captured using cell^D software (Olympus). Virus growth kinetics was assayed by plaque assays in Madin- Darby Canine Kidney (MDCK, ATCC CCL-34) cells at different time points as described previously [56]. All experiments were done in triplicate.

### Ethics statement

Horse tracheas were aseptically collected upon euthanasia from healthy Welsh Mountain ponies and prepared as previously described [56–58]. Animal work was approved by the Ethics Committee of the School of Veterinary Medicine of the University of Glasgow (ethics approval R25A/12). As no regulated procedures were carried out on animals a Home Office license was not required. Embryonated eggs (9–12 days old) were sourced from the State Central Veterinary Laboratory's farm, where they are specifically produced for virus isolation. For this reason no ethics is required by SCVL. In addition, as embryos were killed before the start of the final third of the incubation period, our virus isolation protocol is not considered a regulated procedure under the UK's Animal Scientific Procedures Act 1986. Our study protocols adhered to the UK's Animal Scientific Procedures Act 1986 or to any nationally or internationally recognized guidelines.

## Supporting information

S1 Fig Phylogenetic relationship of the MP genes derived from AIVs isolated from wild birds in Mongolia between 2009 and 2011. Maximum likelihood tree using a sequence dataset comprising 860 IAV sequences representing 21,277 IAV genomes. EIV/Jilin/89 is marked in the phylogeny with a red circle, AIVs isolated in Mongolia (AIVs/2009-11) are indicated with blue and green circles (the latter represent the isolates used in HA assays) and the currently circulating H3N8 EIV lineage (“Classical EIV”) is indicated with a red box. Some branches have been collapsed and appear as triangles for clarity. The scale bar represents the number of substitutions per site. Bootstrap values are indicated next to relevant nodes.
(PDF)

S2 Fig A. Phylogenetic relationship of the PA genes derived from AIVs isolated from wild birds in Mongolia between 2009 and 2011. Maximum likelihood tree using a sequence dataset comprising 860 IAV sequences representing 21,277 IAV genomes. EIV/Jilin/89 is marked in the phylogeny with a red circle, AIVs isolated in Mongolia (AIVs/2009-11) are indicated with blue and green circles (the latter represent the isolates used in HA assays) and the currently circulating H3N8 EIV lineage (“Classical EIV”) is indicated with a red box. Some branches have been collapsed and appear as triangles for clarity. The scale bar represents the number of substitutions per site. Bootstrap values are indicated next to relevant nodes.
(PDF)

S3 Fig A. Phylogenetic relationship of the NS genes derived from AIVs isolated from wild birds in Mongolia between 2009 and 2011. Maximum likelihood tree using a sequence dataset comprising 860 IAV sequences representing 21,277 IAV genomes. EIV/Jilin/89 is marked in the phylogeny with a red circle, AIVs isolated in Mongolia (AIVs/2009-11) are indicated with blue and green circles (the latter represent the isolates used in HA assays) and the currently circulating H3N8 EIV lineage (“Classical EIV”) is indicated with a red box. Some branches have been collapsed and appear as triangles for clarity. The scale bar represents the number of substitutions per site. Bootstrap values are indicated next to relevant nodes.
(PDF)

S4 Fig A. Phylogenetic relationship of the PB2 genes derived from AIVs isolated from wild birds in Mongolia between 2009 and 2011. Maximum likelihood tree using a sequence dataset comprising 860 IAV sequences representing 21,277 IAV genomes. EIV/Jilin/89 is marked in the phylogeny with a red circle, AIVs isolated in Mongolia (AIVs/2009-11) are indicated with blue and green circles (the latter represent the isolates used in HA assays) and the currently circulating H3N8 EIV lineage (“Classical EIV”) is indicated with a red box. Some branches have been collapsed and appear as triangles for clarity. The scale bar represents the number of substitutions per site. Bootstrap values are indicated next to relevant nodes.
(PDF)

S5 Fig A. Phylogenetic relationship of the PB1 genes derived from AIVs isolated from wild birds in Mongolia between 2009 and 2011. Maximum likelihood tree using a sequence dataset comprising 860 IAV sequences representing 21,277 IAV genomes. EIV/Jilin/89 is marked in the phylogeny with a red circle, AIVs isolated in Mongolia (AIVs/2009-11) are indicated with blue and green circles (the latter represent the isolates used in HA assays) and the currently circulating H3N8 EIV lineage (“Classical EIV”) is indicated with a red box. Some branches have been collapsed and appear as triangles for clarity. The scale bar represents the number of substitutions per site. Bootstrap values are indicated next to relevant nodes.
(PDF)

S6 Fig A. Phylogenetic relationship of the NP genes derived from AIVs isolated from wild birds in Mongolia between 2009 and 2011. Maximum likelihood tree using a sequence dataset comprising 860 IAV sequences representing 21,277 IAV genomes. EIV/Jilin/89 is marked in the phylogeny with a red circle, AIVs isolated in Mongolia (AIVs/2009-11) are indicated with blue and green circles (the latter represent the isolates used in HA assays) and the currently circulating H3N8 EIV lineage (“Classical EIV”) is indicated with a red box. Some branches have been collapsed and appear as triangles for clarity. The scale bar represents the number of substitutions per site. Bootstrap values are indicated next to relevant nodes.
(PDF)

S1 Appendix Comparison between hemagglutination inhibition and microneutralization assays. (PDF)

S1 Table Avian influenza viruses isolated and sequenced for this study. (DOCX)

S2 Table Individual hemagglutination inhibition results for viruses used in this study. (XLSX)

S3 Table Equine influenza viruses isolated and sequenced in this study. (XLS)
