# Supplementary figures and images for: Absence of adaptive evolution is the main barrier against influenza emergence in horses in Asia despite frequent virus interspecies transmission from wild birds
Source: PLoS Pathog. 2019 Feb 7;15(2):e1007531. doi: 10.1371/journal.ppat.1007531 (PMC6366691; doi:10.1371/journal.ppat.1007531)

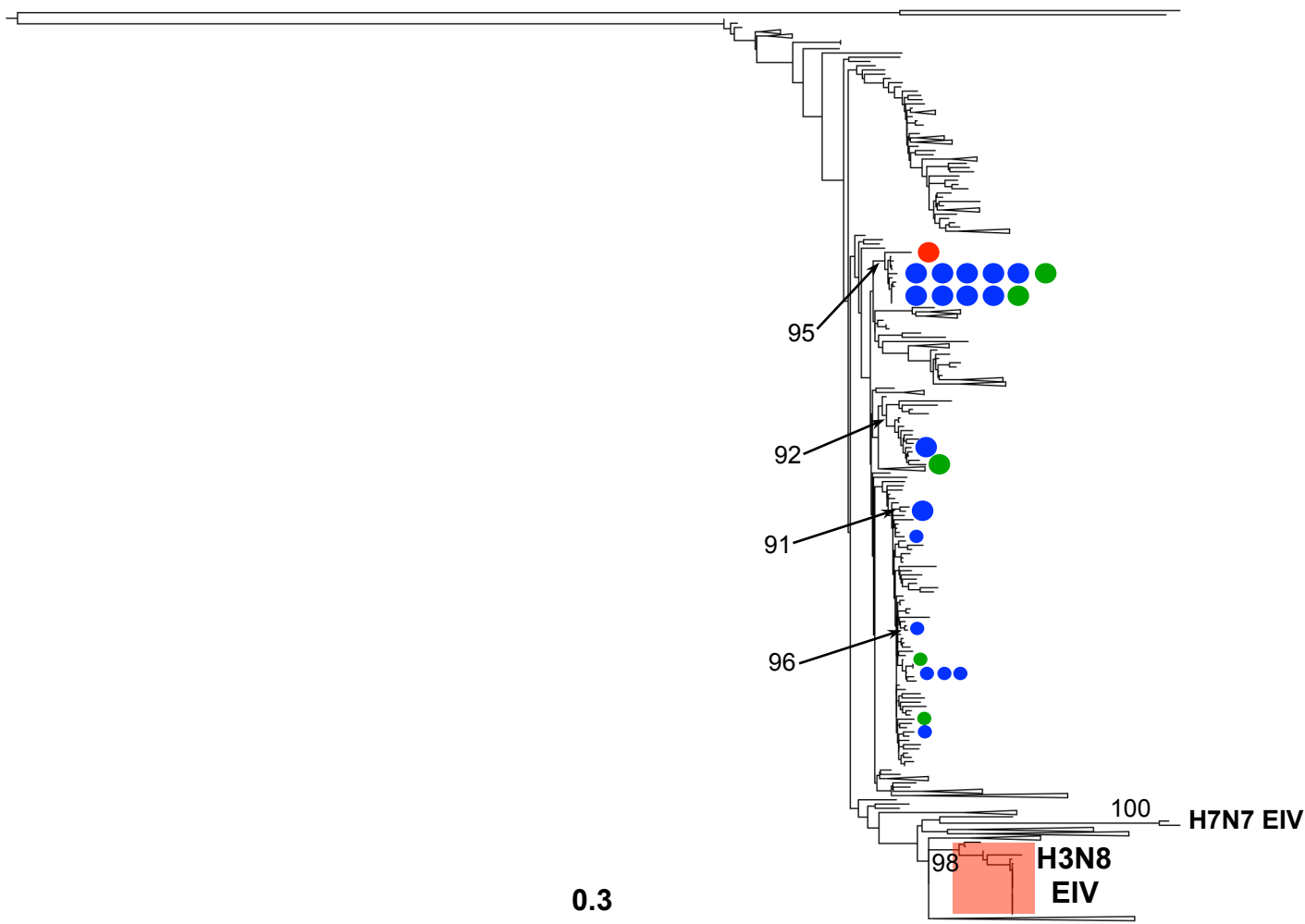

MP

- EIV/Jilin/89
- AIVs collected in Mongolia
- AIVs collected in Mongolia used in serology

Supplement: S1 Fig — Maximum likelihood tree using a sequence dataset comprising 860 IAV sequences representing 21,277 IAV genomes. EIV/Jilin/89 is marked in the phylogeny with a red circle, AIVs isolated in Mongolia (AIVs/2009-11) are indicated with blue and green circles (the latter represent the isolates used in HA assays) and the currently circulating H3N8 EIV lineage (“Classical EIV”) is indicated with a red box. Some branches have been collapsed and appear as triangles for clarity. The scale bar represents the number of substitutions per site. Bootstrap values are indicated next to relevant nodes. (PDF) [file ppat.1007531.s001.pdf]

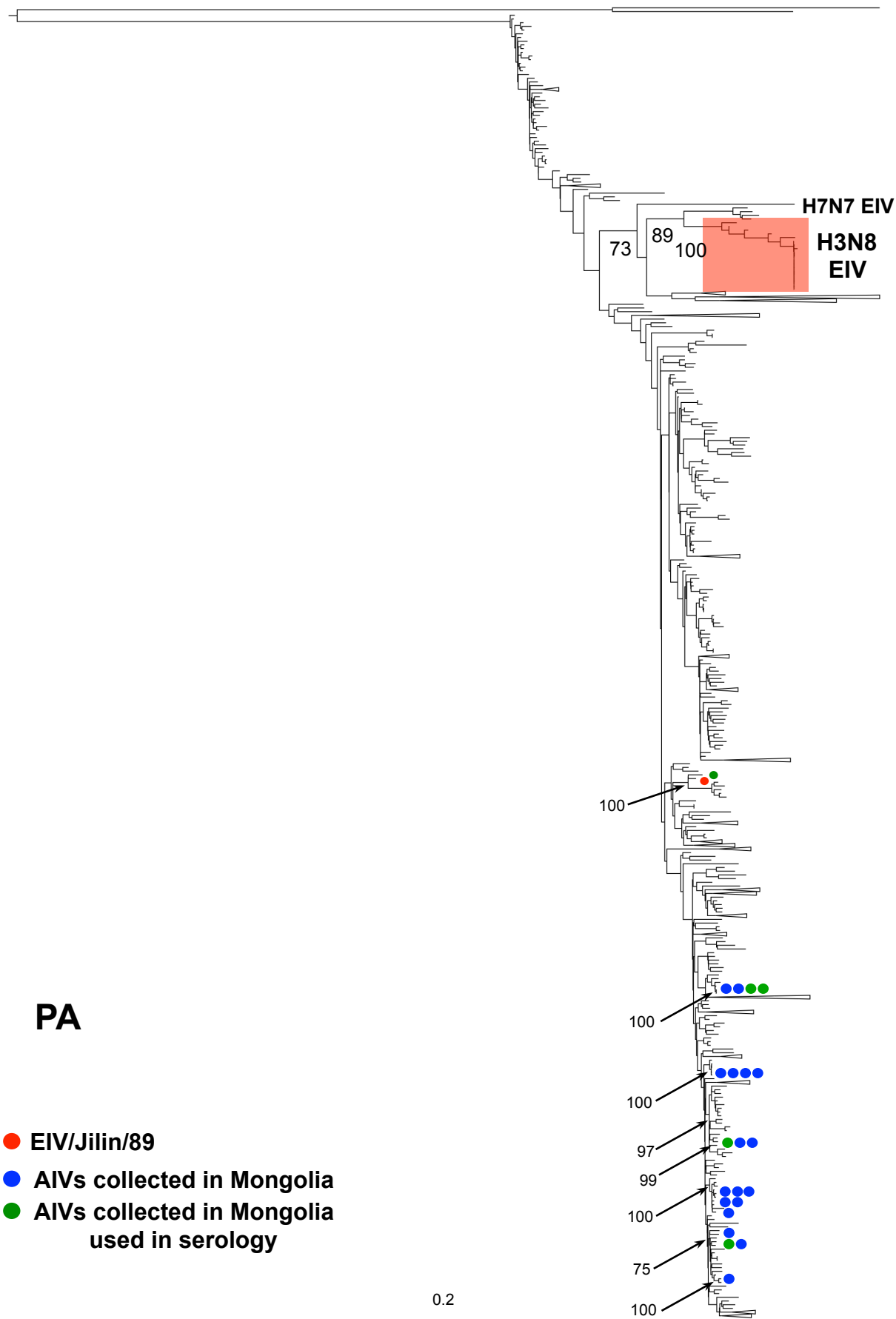

**S2 Figure**

Supplement: S2 Fig — A. Phylogenetic relationship of the PA genes derived from AIVs isolated from wild birds in Mongolia between 2009 and 2011. Maximum likelihood tree using a sequence dataset comprising 860 IAV sequences representing 21,277 IAV genomes. EIV/Jilin/89 is marked in the phylogeny with a red circle, AIVs isolated in Mongolia (AIVs/2009-11) are indicated with blue and green circles (the latter represent the isolates used in HA assays) and the currently circulating H3N8 EIV lineage (“Classical EIV”) is indicated with a red box. Some branches have been collapsed and appear as triangles for clarity. The scale bar represents the number of substitutions per site. Bootstrap values are indicated next to relevant nodes. (PDF) [file ppat.1007531.s002.pdf]

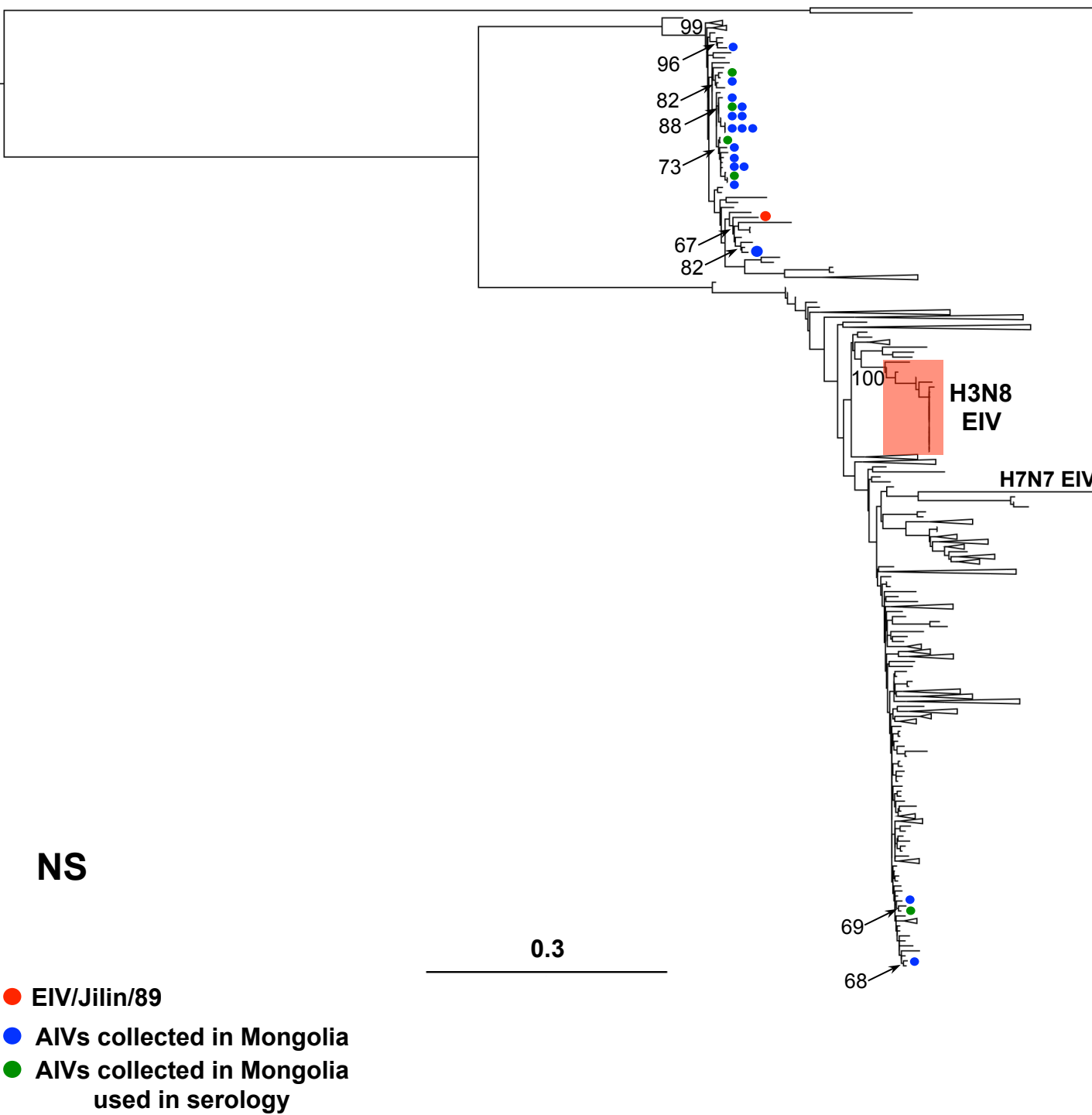

**S3 Figure**

Supplement: S3 Fig — A. Phylogenetic relationship of the NS genes derived from AIVs isolated from wild birds in Mongolia between 2009 and 2011. Maximum likelihood tree using a sequence dataset comprising 860 IAV sequences representing 21,277 IAV genomes. EIV/Jilin/89 is marked in the phylogeny with a red circle, AIVs isolated in Mongolia (AIVs/2009-11) are indicated with blue and green circles (the latter represent the isolates used in HA assays) and the currently circulating H3N8 EIV lineage (“Classical EIV”) is indicated with a red box. Some branches have been collapsed and appear as triangles for clarity. The scale bar represents the number of substitutions per site. Bootstrap values are indicated next to relevant nodes. (PDF) [file ppat.1007531.s003.pdf]

**PB2**

- EIV/Jilin/89
- AIVs collected in Mongolia
- AIVs collected in Mongolia used in serology

0.2

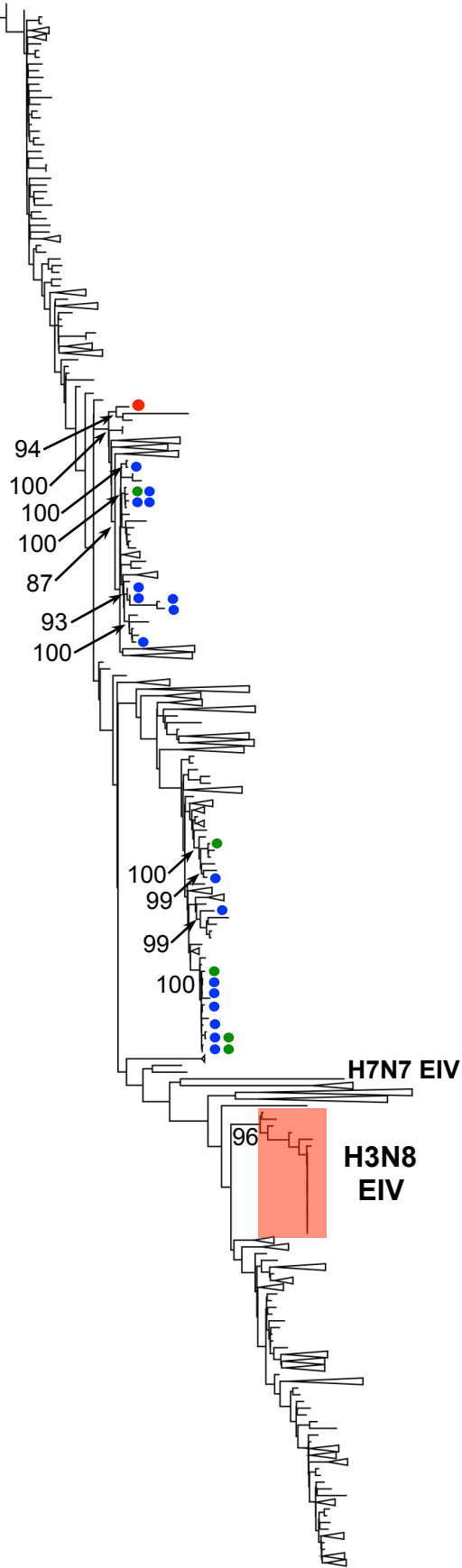

Supplement: S4 Fig — A. Phylogenetic relationship of the PB2 genes derived from AIVs isolated from wild birds in Mongolia between 2009 and 2011. Maximum likelihood tree using a sequence dataset comprising 860 IAV sequences representing 21,277 IAV genomes. EIV/Jilin/89 is marked in the phylogeny with a red circle, AIVs isolated in Mongolia (AIVs/2009-11) are indicated with blue and green circles (the latter represent the isolates used in HA assays) and the currently circulating H3N8 EIV lineage (“Classical EIV”) is indicated with a red box. Some branches have been collapsed and appear as triangles for clarity. The scale bar represents the number of substitutions per site. Bootstrap values are indicated next to relevant nodes. (PDF) [file ppat.1007531.s004.pdf]

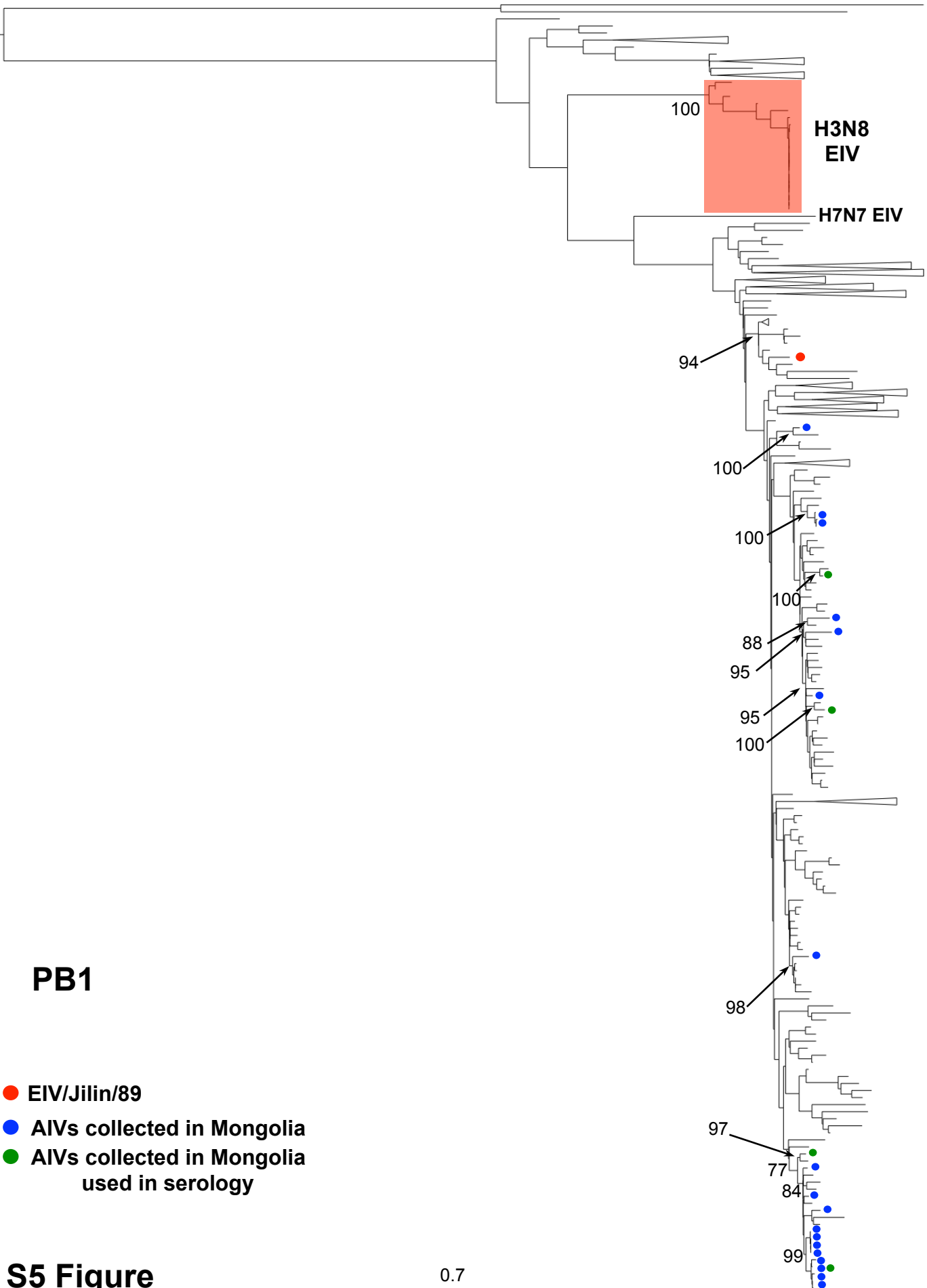

Supplement: S5 Fig — A. Phylogenetic relationship of the PB1 genes derived from AIVs isolated from wild birds in Mongolia between 2009 and 2011. Maximum likelihood tree using a sequence dataset comprising 860 IAV sequences representing 21,277 IAV genomes. EIV/Jilin/89 is marked in the phylogeny with a red circle, AIVs isolated in Mongolia (AIVs/2009-11) are indicated with blue and green circles (the latter represent the isolates used in HA assays) and the currently circulating H3N8 EIV lineage (“Classical EIV”) is indicated with a red box. Some branches have been collapsed and appear as triangles for clarity. The scale bar represents the number of substitutions per site. Bootstrap values are indicated next to relevant nodes. (PDF) [file ppat.1007531.s005.pdf]

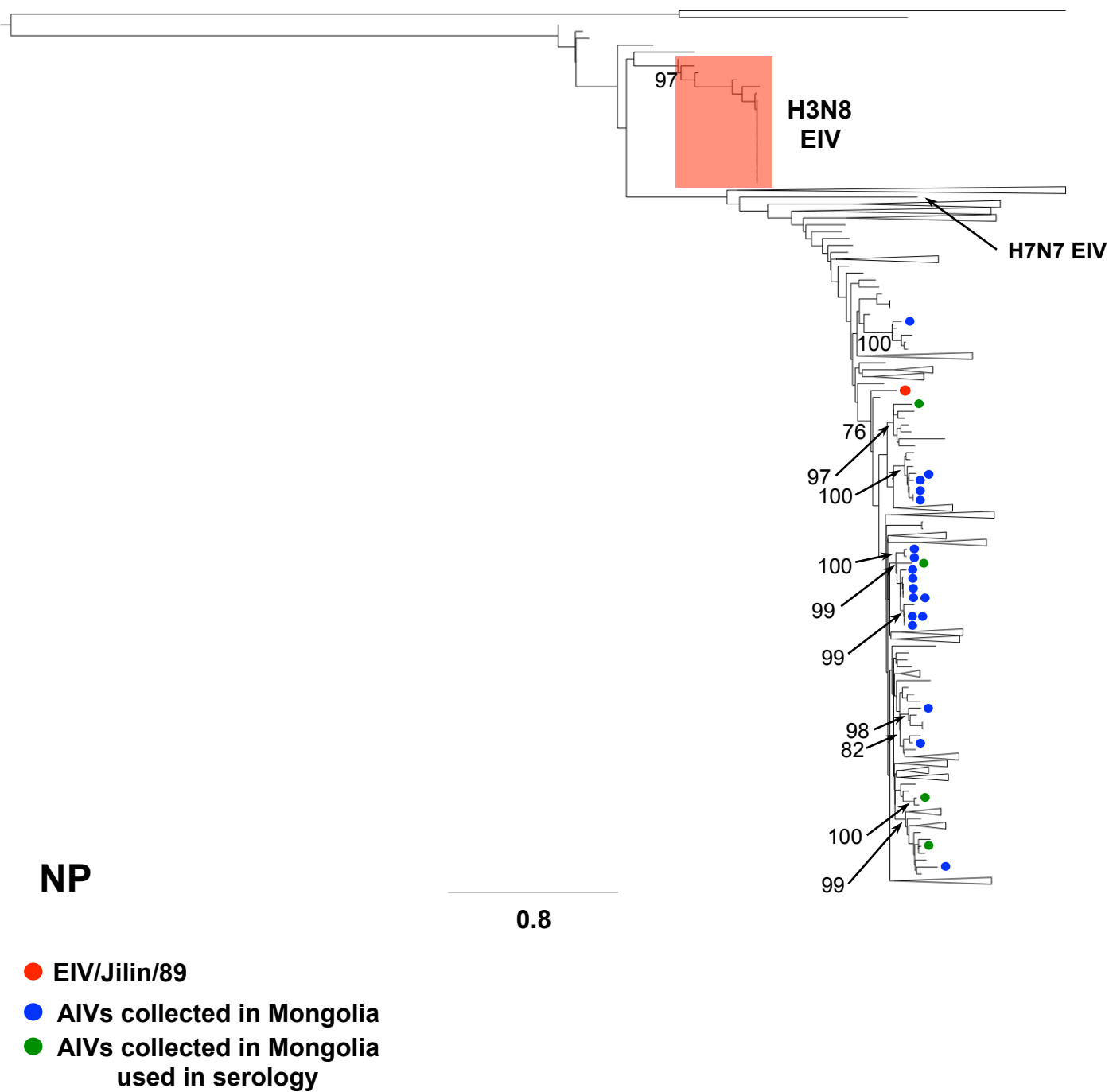

**S6 Figure**

Supplement: S6 Fig — A. Phylogenetic relationship of the NP genes derived from AIVs isolated from wild birds in Mongolia between 2009 and 2011. Maximum likelihood tree using a sequence dataset comprising 860 IAV sequences representing 21,277 IAV genomes. EIV/Jilin/89 is marked in the phylogeny with a red circle, AIVs isolated in Mongolia (AIVs/2009-11) are indicated with blue and green circles (the latter represent the isolates used in HA assays) and the currently circulating H3N8 EIV lineage (“Classical EIV”) is indicated with a red box. Some branches have been collapsed and appear as triangles for clarity. The scale bar represents the number of substitutions per site. Bootstrap values are indicated next to relevant nodes. (PDF) [file ppat.1007531.s006.pdf]
