# Supplementary material for: Absence of adaptive evolution is the main barrier against influenza emergence in horses in Asia despite frequent virus interspecies transmission from wild birds
Source: PLoS Pathog. 2019 Feb 7;15(2):e1007531. doi: 10.1371/journal.ppat.1007531 (PMC6366691; doi:10.1371/journal.ppat.1007531)
Supplement: S1 Appendix — (PDF) [file ppat.1007531.s007.pdf]

S1 Appendix. Comparison between hemagglutination inhibition and microneutralization assays.

Horse serum samples that were positive by hemagglutination inhibition (HI) to the avian influenza viruses under study (AIV/881, AIV/963, AIV/2076, AIV/2106, and AIV/2271) were further tested in microneutralization (MN) assays following standard protocols available in the *Manual for the laboratory diagnosis and virological surveillance of influenza* (WHO Global Influenza Surveillance Network, 2011). As some serum samples were cytotoxic, a lower number of sera was tested in MN assays for all viruses except AIV/963. The table below shows that HI and MN results shared ≥83.3% consistency. On average, 91.7% of serum samples positive to AIVs by HI were also positive in MN assays.

Virus	HI[a]	MN[b]	Agreement (%)
**AIV/881**	33(38)	32	97
**AIV/963**	74(74)	69	93.2
**AIV/2076**	34(36)	31	91.2
**AIV/2106**	34(36)	32	94.1
**AIV/2271**	36(44)	30	83.3

[a] Number of positive serum samples tested using the HI assay. Between brackets is shown the total number of HI positive samples.
[b] Number of positive serum samples tested using the MN assay.
