# Supplementary material for: Absence of adaptive evolution is the main barrier against influenza emergence in horses in Asia despite frequent virus interspecies transmission from wild birds
Source: PLoS Pathog. 2019 Feb 7;15(2):e1007531. doi: 10.1371/journal.ppat.1007531 (PMC6366691; doi:10.1371/journal.ppat.1007531)
Supplement: S1 Table — (DOCX) [file ppat.1007531.s008.docx]

**Supplementary 1 Table. Avian influenza viruses isolated and sequenced for this study.**

| **Abbreviation*** | **Virus name** | **Taxonomy number**** | **Subtype** | **Genome coverage***** | **Geographical**  **Longitude** | **Geographical**  **Latitude** | **Collection date** |
| --- | --- | --- | --- | --- | --- | --- | --- |
|  | A/northern shoveler/Mongolia/992V/2009 | 1316855 | H3N8 | C | 101.1633 | 49.05795 | 2009-09-15 |
| AIV/2271 | A/mallard/Mongolia/2377/2011 | 1316865 | H3N6 | C | 113.97216 | 45.20491 | 11-10-2011 |
|  | A/red-crested pochard/Mongolia/463V/2009 | 1316868 | H3N1 | C | 93.34398 | 48.85717 | 2009-08-17 |
|  | A/common teal/Mongolia/2271/2011 | 1316850 | H3N8 | P | 114.12471 | 45.25129 | 10-10-2011 |
|  | A/common shelduck/Mongolia/2185/2011 | 1316848 | H3N8 | C | 113.81112 | 45.30478 | 09-10-2011 |
|  | A/common shelduck/Mongolia/2157/2011 | 1316847 | H3N3 | C | 113.81112 | 45.30478 | 09-10-2011 |
| AIV/2106 | A/common shelduck/Mongolia/2106/2011 | 1316846 | H3N8 | C | 113.81112 | 45.30478 | 09-10-2011 |
| AIV/2076 | A/common shelduck/Mongolia/2076/2011 | 1316845 | H3N8 | C | 113.99162 | 45.26889 | 08-10-2011 |
|  | A/ruddy shelduck/Mongolia/1787/2011 | 1316863 | H3N8 | C | 113.97216 | 45.20491 | 17-09-2011 |
|  | A/wild bird/Mongolia/1782/2011 | 1382831 | H3N8 | P | 113.97216 | 45.20491 | 17-09-2011 |
|  | A/common teal/Mongolia/1906/2011 | 1316849 | H3N8 | C | 114.12471 | 45.25129 | 18-09-2011 |
|  | A/mallard/Mongolia/1581/2010 | 1316852 | H3N8 | C | 113.81112 | 45.30478 | 2010-09-10 |
|  | A/mallard /Mongolia/1551/2010 | 1316867 | H3N1 | C | 113.81112 | 45.30478 | 2010-09-10 |
|  | A/northern shoveler/Mongolia/977V/2009 | 1316854 | H3N8 | P | 101.1633 | 49.05795 | 2009-09-15 |
|  | A/northern shoveler/Mongolia/973/2009 | 1316851 | H3N8 | C | 101.1633 | 49.05795 | 2009-09-15 |
|  | A/velvet scoter/Mongolia/969V/2009 | 1316864 | H3N8 | C | 101.1633 | 49.05795 | 2009-09-15 |
| AIV/963 | A/ruddy shelduck/Mongolia/963V/2009 | 1316888 | H3N8 | C | 101.1633 | 49.05795 | 2009-09-15 |
|  | A/ruddy shelduck/Mongolia/961V/2009 | 1316887 | H3N8 | C | 101.1633 | 49.05795 | 2009-09-15 |
|  | A/northern shoveler/Mongolia/957/2009 | 1316853 | H3N8 | P | 101.1633 | 49.05795 | 2009-09-15 |
|  | A/northern shoveler/Mongolia/899V/2009 | 1316866 | H3N6 | P | 101.1633 | 49.05795 | 2009-09-15 |
|  | A/ruddy shelduck/Mongolia/882V/2009 | 1316857 | H3N8 | C | 101.1633 | 49.05795 | 2009-09-15 |
| AIV/881 | A/ruddy shelduck/Mongolia/881V/2009 | 1316856 | H3N8 | C | 101.1633 | 49.05795 | 2009-09-15 |

* Abbreviated names of viruses used in serological assays and experimental infections.

** https://www.ncbi.nlm.nih.gov/taxonomy

*** P= Partial; C= Complete.
